# Help-seeking following a flooding event: a cross-sectional analysis of adults affected by flooding in England in winter 2013/14

**DOI:** 10.1093/eurpub/ckad082

**Published:** 2023-06-16

**Authors:** L Findlater, C Robin, K Hopgood, T Waite, National Study of Flooding and Health Study Group, Thomas Waite, Charles Beck, Isabel Oliver, Richard Amlôt, Angie Bone, Giovanni Leonardi, Gideon James Rubin, Sari Kovats, Ben Armstrong, G Rubin, C R Beck, I Oliver

**Affiliations:** UK Health Security Agency, Bristol, UK; National Institute of Health Research (NIHR) Health Protection Research Unit (HPRU) on Behavioural Science and Evaluation at the University of Bristol, Bristol, UK; UK Health Security Agency, Bristol, UK; National Institute of Health Research (NIHR) Health Protection Research Unit (HPRU) on Behavioural Science and Evaluation at the University of Bristol, Bristol, UK; UK Health Security Agency, Bristol, UK; Department of Health and Social Care, UK; King’s College London, London, UK; NIHR HPRU on Emergency Preparedness and Response at King’s College London, London, UK; UK Health Security Agency, Bristol, UK; National Institute of Health Research (NIHR) Health Protection Research Unit (HPRU) on Behavioural Science and Evaluation at the University of Bristol, Bristol, UK; UK Health Security Agency, Bristol, UK; National Institute of Health Research (NIHR) Health Protection Research Unit (HPRU) on Behavioural Science and Evaluation at the University of Bristol, Bristol, UK

## Abstract

**Background:**

Flooding can cause long-term, significant impacts on mental health in affected populations. We explored help-seeking behaviour of households affected by flooding.

**Methods:**

A cross-sectional analysis was conducted on National Study of Flooding and Health data on households flooded in England in winter 2013/14. Participants (Year 1: *n* = 2006; Year 2: *n* = 988; Year 3: *n* = 819) were asked if they sought help from health services and other sources. Logistic regression was conducted to calculate odds ratios (ORs) of help-seeking in flooded and disrupted participants compared to unaffected, adjusted for *a priori* confounders.

**Results:**

The odds of seeking help from any source 1 year after flooding were greater for flooded participants [adjusted OR (aOR): 1.71, 95% confidence interval (CI): 1.19–1.45] and those disrupted by flooding (aOR: 1.92, 95% CI: 1.37–2.68) compared to unaffected participants. This continued in the second year (flooded: aOR 6.24, 95% CI: 3.18–13.34; disrupted: aOR: 2.22, 95% CI: 1.14–4.68), and help-seeking remained greater in flooded than unaffected participants in the third year. Flooded and disrupted participants were particularly likely to seek help from informal sources. Help-seeking was more prevalent amongst participants with mental health outcomes, but a notable proportion of individuals with any mental health outcome did not seek help (Year 1: 15.0%; Year 2: 33.3%; Year 3: 40.3%).

**Conclusions:**

Flooding is associated with increased demand for formal and informal support, persisting for at least 3 years, and an unmet need for help amongst affected individuals. Our findings should be considered in flood response planning to reduce the long-term adverse health impacts of flooding.

## Introduction

Flooding is associated with disruption to health and social care services and a short-term increased risk of injury, death, infectious disease and exacerbation of existing health conditions.^1^ In more economically developed countries, the short-term physical health effects of flooding are typically well mitigated; however, there is a large and long-term impact on mental health.^2–5^ Several studies have found that flooding of the home directly increases the risk of psychological morbidity, including symptoms associated with anxiety, depression, and post-traumatic stress disorder (PTSD).^5–8^ The English National Study of Flooding and Health has demonstrated that the prevalence of probable psychological morbidity is high among people affected by flooding and that the increased risk persists up to 3 years after flooding.^3–5^

Few studies have explored the impact of flooding on help-seeking behaviours particularly beyond the immediate period of flooding. Help-seeking to obtain external assistance and support about a mental health or emotional concern, from formal sources (e.g. a healthcare professional) or informal sources (e.g. friends and family), is not well understood.^9^^,^^10^ There are no recent data on the impact of flooding on help-seeking behaviours; however, research from 40 years ago, using hospital attendance data, found that hospital use and primary care attendance increased following flooding.^11^ Other research has described that institutional support plays an important role in mental wellbeing after flooding, with interventions such as evacuation and flood defence works having negative and positive implications for mental health.^12^ Community networks, including local volunteers, donations from local business and informal support from neighbours, have also been described as sources of help and may help to offset the negative impact of flooding on wellbeing.^13^

There is a lack of evidence regarding the impact of flooding on the use of health services for psychological morbidity. However, research has shown that people with psychological morbidity frequently do not seek help from professional support services; the preferred source of help is often informal support from family and friends rather than formal health services from a GP or counsellor.^10^^,^^14^^,^^15^ After a flooding event, existing support structures are not always accessed by those in need, especially for mental health issues.^12^ The delay in seeking timely clinical help when needed can result in disability, suicide, lowered quality of life and impaired physical and social functioning, even for those with less severe mental ill-health.^16^^,^^17^

It is projected that if no preventive measures are taken, flooding may affect 252 000–480 000 additional people per year across Europe by the 2080s.^18–20^ In the UK, climate change is projected to increase the number of properties at risk of flooding from all sources and including in areas that have not previously been at risk of flooding, whilst housing need and economic growth requiring more development are also projected to exacerbate flood risk.^21^ It is therefore important to understand the effect that flooding has on help-seeking behaviours in order to enable services, including the voluntary sector, to plan and react to the needs of those indirectly or directly affected by flooding through climate change adaptation plans and flood response plans.^22^

This article describes and explores help-seeking behaviours from a broad range of services (including GP services, mental health services, hospitals, NHS 111, charities and family and friends) for up to 3 years after the 2013/14 floods in England, to determine if flooding is associated with help-seeking behaviour and which factors are associated with this behaviour.

## Methods

### Setting and population

This cross-sectional analysis is based on self-completed questionnaires sent in January 2015—8761 households in postcode areas known to have been affected by flooding between 1 December 2013 and 31 March 2014 as part of the National Study of Flooding and Health. All adults resident at households sent a study pack, including those which did not experience flooding, were eligible to participate by returning the questionnaire either by paper or online. Individuals who consented to follow-up over subsequent years were sent questionnaires to self-complete in January 2016 (*n* = 1408) and January 2017 (*n* = 1361), 2 and 3 years after the flooding event. Full descriptions of participant recruitment, characteristics and sample size have been described previously.^3–5^^,^^23–26^

### Data collection

The 36-item questionnaire sent in the first year after flooding included questions assessing demographic variables, exposure to flooding, disruption to health and social care services, longstanding limiting illness, validated screening instruments for depression, anxiety and PTSD, and two questions on help-seeking behaviour (Supplementary figure SA1).^27–29^ An ‘any probable mental health outcome’ variable was created to indicate if participants screened positive for probable depression, anxiety and/or PTSD using the validated screening instruments.

People who reported flood water in liveable rooms of their home were defined as ‘flooded’, people who reported disruption to their lives as a result of flooding (e.g. loss of access to work or services) in the absence of flood water in liveable rooms of their home were defined as ‘disrupted’, and people who reported no impact of any kind as a result of flooding were defined as ‘unaffected’.^3–5^

In the first year, participants were asked if they had seen a doctor, counsellor or health professional or received help from the sources listed (GP, hospital, NHS 111, therapist/counsellor, family/friends, voluntary, none) either since 1 December 2013 or in the last 4 weeks. Participants could select multiple responses. In the second and third years, the questionnaire asked whether participants had sought or received treatment for any stress-related, mental or emotional problem since the 2013/14 flooding from the sources listed (GP, practice nurse, mental health services, another health professional, voluntary, family/friends) either (i) in the past year and (ii) in the past 4 weeks (Supplementary figures SA2 and SA3).

### Statistical methods

Descriptive analysis of participant characteristics and exposures was conducted. The primary outcome was help-seeking from any source in the 12 months prior to questionnaire completion, which was described in separate analyses for the first, second and third year after flooding exposure. Sub-group analysis for formal and informal sources of help was also undertaken. When exposure group was unknown, the participant was excluded from analysis.

A secondary outcome of help-seeking from any source in the last 4 weeks was also assessed in the questionnaires. Due to smaller numbers seeking help in the last 4 weeks, help sources were grouped into (i) ‘formal’ help (health services including GP, hospital, NHS 111 and mental health services) and (ii) ‘informal’ help (voluntary/charity services and friends and/or family) for sub-group analysis.

For the second and third year after flooding exposure, there was an addition to the questionnaire, including a question asking participants if they had received treatment from formal or informal sources. This allowed us to describe help-seeking and receiving treatment separately in those years.

Associations between the binary outcomes of help-seeking (yes/no) and exposure to flooding were examined using logistic regression models in STATA V13 (StataCorp, TX, USA).

As with the National Study of Flooding and Health, multiple logistic regression models were used to calculate odds ratios for the flooded and disrupted groups compared to the unaffected group. Adjusted odds ratios were adjusted for socio-economic variables considered *a priori* to be potential confounders from previous literature: age, sex, deprivation, local authority, ethnicity, marital status, reported pre-existing illness, education and employment status.^3–5^^,^^10^^,^^15^^,^^30–32^

### Ethical approval

Ethical approval for the English National Cohort Study of Flooding and Health was granted by the Research Ethics Committee of the School of Psychiatry, Nursing and Midwifery at King’s College London [Reference PNM/13/14-152]. All participants consented to participate in the study.

## Results

### Help-seeking behaviour

In the first year after flooding, the proportion of individuals seeking help from any source in the previous 12 months was greater for those classified as flooded (77.2%) or disrupted (78.3%) than for those unaffected by flooding (67.5%) (table 1). Help-seeking behaviour in the 4 weeks prior to questionnaire completion was also greater for flooded individuals (47.1%), compared with disrupted (44.2%) or unaffected (38.9%) individuals. This trend persisted 2 years after flooding, with help-seeking from any source in the previous 12 months for stress-related, mental or emotional problems being more frequent in flooded (36.5%) or disrupted (20.0%) individuals compared to those unaffected (9.7%); this was also seen in the third year after flooding (31.7% of flooded individuals sought any help, compared with 21.4% of disrupted and 14.7% of unaffected individuals). Whilst overall help-seeking behaviour decreased with each year after the flooding event, more individuals sought help from formal than informal sources each year (table 1, Supplementary table SA1).

**Table 1:** Number of individuals who sought help by exposure status (Years 1–3) [*n* (%)]

Outcome	Unaffected	Disrupted	Flooded	Overall
**Sought help**				

**Year 1**	***n* = 285**	***n* = 1099**	***n* = 622**	***n* = 2006**
Since 1 December 2013				
Any help	189/280 (67.5)	830/1060 (78.3)	464/601 (77.2)	1483/1941 (76.4)
Formal source^a^	185/280 (66.1)	790/1060 (74.5)	429/601 (71.4)	1404/1941 (72.3)
Informal source^a^	37/280 (13.2)	256/1060 (24.2)	206/601 (34.3)	499/1941 (25.7)
In the last 4 weeks				
Any help	109/280 (38.9)	469/1060 (44.2)	283/601 (47.1)	861/1941 (44.4)
Formal source^a^	95/280 (33.9)	406/1060 (38.3)	237/601 (39.4)	738/1941 (38.0)
Informal source^a^	25/280 (8.9)	155/1060 (14.6)	118/601 (19.6)	298/1941 (15.4)

**Year 2**	***n* = 137**	***n* = 512**	***n* = 339**	***n* = 988**
In the last year				
Any help	13/134 (9.7)	96/479 (20.0)	118/323 (36.5)	227/936 (24.3)
Formal source^b^	12/134 (9.0)	74/477 (15.5)	86/323 (26.6)	172/934 (18.4)
Informal source^b^	7/131 (5.3)	51/467 (10.9)	68/298 (22.8)	126/896 (14.1)
In the last 4 weeks				
Any help	4/131 (3.1)	36/461 (7.8)	39/295 (13.2)	79/887 (8.9)
Formal^a^	3/131 (2.3)	25/461 (5.4)	26/298 (8.7)	54/890 (6.1)
Informal^a^	3/131 (2.3)	20/461 (4.3)	23/293 (7.8)	46/885 (5.2)

**Year 3**	***n* = 119**	***n* = 421**	***n* = 279**	***n* = 819**
In the last year				
Any help	17/116 (14.7)	86/402 (21.4)	85/268 (31.7)	188/786 (23.9)
Formal^b^	13/116 (11.2)	60/400 (15.0)	69/268 (25.7)	142/784 (18.1)
Informal^b^	8/114 (7.0)	48/389 (12.3)	46/252 (18.3)	102/755 (13.5)
In the last 4 weeks				
Any help	8/114 (7.0)	36/387 (9.3)	38/252 (15.1)	82/711 (11.5)
Formal^b^	4/114 (3.5)	24/387 (6.2)	27/251 (10.8)	55/752 (7.3)
Informal^b^	6/114 (5.3)	22/386 (5.7)	24/251 (9.6)	52/751 (6.9)

a For Year 1: formal source includes GP, hospital, therapist, NHS 111; informal source includes voluntary/charity and family/friends. Help-seeking related to any reason or condition.

b For Years 2 and 3: formal source includes GP, nurse, mental health services and other health professional; informal source includes voluntary groups and family/friends. Help-seeking related to ‘stress-related, mental or emotional problem[s]’ only.

### Receiving treatment

A greater proportion of flooded and disrupted individuals received help than unaffected individuals 2 and 3 years after flooding (table 2, Supplementary table SA2). In the second year of flooding, 53.2% of flooded individuals who sought help in the past year received any treatment (19.4/36.5, a difference of −17.1%). For disrupted individuals, 65.5% of those who sought help received any treatment (13.1/20.0, a difference of −6.9%); for unaffected individuals, 70.1% of those who sought help received any treatment (6.8/9.7, a difference of −2.9%). In the third year of flooding, 47.0% of flooded individuals who sought help in the past year received any treatment (14.9/31.7, a difference of −16.8%); 38.8% of disrupted individuals who sought help received treatment (8.3/21.4, a difference of −13.1%); and 35.4% of unaffected individuals who sought help received treatment (5.2/14.7, a difference of −9.5%).

**Table 2:** Number of individuals who received treatment (Years 2 and 3) [*n* (%)]

Outcome	Unaffected	Disrupted	Flooded	Overall
**Received treatment**				

**Year 2**	***n* = 137**	***n* = 512**	***n* = 339**	***n* = 988**
In the last year				
Any help	9/132 (6.8)	63/482 (13.1)	61/314 (19.4)	133/928 (14.3)
Formal source^a^	9/132 (6.8)	59/482 (12.2)	58/315 (18.4)	126/929 (13.6)
Informal source^a^	2/130 (1.5)	9/468 (1.9)	8/295 (2.7)	19/893 (2.1)
In the last 4 weeks				
Any help	3/130 (2.3)	23/470 (4.9)	19/299 (6.4)	45/899 (5.0)
Formal	3/130 (2.3)	19/471 (4.0)	18/300 (6.0)	40/901 (4.4)
Informal	1/130 (0.8)	4/468 (0.9)	2/295 (0.7)	7/893 (0.8)

**Year 3**	***n* = 119**	***n* = 421**	***n* = 279**	***n* = 819**
In the last year				
Any help	6/115 (5.2)	33/398 (8.3)	39/262 (14.9)	109/775 (14.1)
Formal	10/115 (8.7)	45/399 (11.3)	52/262 (19.8)	107/776 (13.8)
Informal	1/114 (0.9)	5/390 (1.3)	6/250 (2.4)	12/754 (1.6)
In the last 4 weeks				
Any help	5/114 (4.4)	17/389 (4.4)	20/250 (8.0)	42/753 (5.6)
Formal	5/114 (4.4)	17/390 (4.4)	19/250 (7.6)	41/754 (5.4)
Informal	1/114 (0.9)	1/390 (0.3)	3/250 (1.2)	5/754 (0.7)

a For Years 2 and 3, formal source includes GP, nurse, mental health services and other health professional; informal source includes voluntary groups and family/friends.

### Mental health outcomes and help-seeking behaviour

Help-seeking behaviour from any source was more prevalent in individuals with any mental health outcome (i.e. probable depression, anxiety or PTSD) (85.0%) than those without a mental health outcome (65.4%), both in the past year, and in the preceding 4 weeks (table 3). This difference in help-seeking was greater in Year 2 after flooding, with 66.7% of those with a mental health condition seeking help in the past 12 months compared to 14.2% of those without a mental health condition; this persisted in Year 3 (help-seeking: 59.7% in those with and 14.7% in those without a mental health condition). A notable proportion of individuals with any mental health outcome did not reporting seeking help from any source in the past year; this was 15.0% in the first year, 33.3% in the second year; and 40.3% in the third year (table 3).

**Table 3:** Proportion of all respondents seeking help from formal and informal sources, by mental health outcome (Years 1–3)

	Last 4 weeks			Last 12 months		
Mental health status	Any source	Formal source**^a^**	Informal source**^a^**	Any source	Formal source**^a^**	Informal source**^a^**
	*n* (%)	*n* (%)	*n* (%)	*n* (%)	*n* (%)	*n* (%)
Year 1						
Any mental health outcome (*n* = 519)	292 (56.3)	251 (48.4)	129 (24.9)	441 (85.0)	410 (79.0)	207 (39.9)
Probable anxiety (*n* = 299)	176 (58.9)	147 (49.2)	86 (28.8)	253 (84.6)	234 (78.3)	130 (43.5)
Probable depression (*n* = 239)	150 (62.8)	133 (55.6)	69 (28.9)	205 (85.8)	194 (81.2)	98 (41.0)
Probable PTSD (*n* = 392)	231 (58.9)	198 (50.5)	106 (27.0)	332 (84.7)	309 (78.8)	163 (41.6)
No mental health outcome (*n* = 1357)	543 (40.0)	465 (34.3)	162 (11.9)	991 (73.0)	945 (69.6)	277 (20.4)
Year 2						
Any mental health outcome (*n* = 162)	45/136 (33.1)	32/136 (23.5)	27/137 (19.7)	104/156 (66.7)	81/154 (52.6)	60/142 (42.3)
Probable anxiety (*n* = 83)	27/66 (40.9)	19/65 (29.2)	17/66 (25.8)	56/80 (70.0)	47/78 (60.3)	29/69 (42.0)
Probable depression (*n* = 59)	17/47 (36.2)	12/46 (26.1)	12/46 (26.1)	40/57 (70.2)	37/57 (64.9)	22/47 (46.8)
Probable PTSD (*n* = 129)	37/105 (35.2)	26/106 (24.5)	23/109 (21.1)	85/125 (68.0)	65/124 (52.4)	50/113 (44.2)
No mental health outcome (*n* = 702)	24/664 (3.6)	12/667 (1.8)	15/666 (2.3)	97/682 (14.2)	67/683 (9.8)	55/668 (8.2)
Year 3						
Any mental health outcome (*n* = 121)	31/104 (29.8)	21/103 (20.4)	18/103 (17.5)	71/119 (59.7)	58/118 (49.2)	41/104 (39.4)
Probable anxiety (*n* = 59)	17/50 (34.0)	12/49 (24.5)	11/50 (22.0)	35/59 (59.3)	31/58 (53.4)	22/51 (43.1)
Probable depression (*n* = 42)	12/33 (36.4)	8/32 (25.0)	7/34 (20.6)	32/42 (76.2)	29/42 (69.0)	17/34 (50.0)
Probable PTSD (*n* = 91)	23/76 (30.3)	15/75 (20.0)	15/76 (19.7)	59/89 (66.3)	48/88 (54.5)	36/77 (46.8)
No mental health outcome (*n* = 608)	37/588 (6.3)	24/587 (4.1)	24/588 (4.1)	88/599 (14.7)	61/599 (10.2)	48/589 (8.1)

a For Year 1: formal source includes GP, hospital, therapist, NHS 111; informal source includes voluntary/charity and family/friends. For Years 2 and 3: formal source includes GP, nurse, mental health services and other health professional; informal source includes voluntary groups and family/friends.

### Regression analysis

When adjusted for *a priori* confounders, the adjusted odds ratio (aOR) of seeking help from any source in the past 12 months was greater for flooded participants [aOR: 1.71, 95% confidence interval (CI): 1.19–2.45] and for those disrupted by flooding (aOR: 1.92, 95% CI: 1.37–2.68) in the first year after flooding (table 4). The adjusted odds of seeking help from any source in the four weeks preceding questionnaire completion were also greater for flooded participants (aOR: 1.50, 95% CI: 1.08–2.10) and for those disrupted by flooding (aOR: 1.24, 95% CI: 0.98–1.83) than those unaffected.

**Table 4:** Crude and adjusted odds ratios (ORs) for overall, formal and informal help-seeking in the 12 months and in the 4 weeks before questionnaire completion (Years 1–3)

		Exposure group	
Outcome	Odds ratio (OR)	Disrupted	Flooded
**Sought help**			
**Year 1**			
Since December 2013			
Any source	Crude OR (95% CI)	1.74 (1.30–2.32)	1.63 (1.19–2.23)
	aOR^c^ (95% CI)	1.92 (1.37–2.68)	1.71 (1.19–2.45)
Formal source^a^	Crude OR (95% CI)	1.50 (1.13–2.00)	1.28 (0.94–1.73)
	aOR (95% CI)	1.69 (1.22–2.34)	1.38 (0.97–1.96)
Informal source^a^	Crude OR (95% CI)	2.09 (1.46–3.08)	3.43 (2.36–5.10)
	aOR (95% CI)	2.47 (1.61–3.91)	4.22 (2.71–6.79)
In last 4 weeks			
Any source	Crude OR (95% CI)	1.24 (0.95–1.63)	1.40 (1.05–1.87)
	aOR (95% CI)	1.24 (0.98–1.83)	1.50 (1.08–2.10)
Formal source	Crude OR (95% CI)	1.21 (0.92–1.60)	1.27 (0.94–1.71)
	aOR (95% CI)	1.33 (0.97–1.83)	1.41 (1.00–1.99)
Informal source	Crude OR (95% CI)	1.75 (1.14–2.78)	2.49 (1.60–4.02)
	aOR (95% CI)	2.25 (1.33–4.02)	3.52 (2.05–6.38)
**Year 2**			
In the last 12 months			
Any source	Crude OR (95% CI)	2.33 (1.30–4.50)	5.36 (2.99–10.35)
	aOR (95% CI)	2.22 (1.14–4.68)	6.24 (3.18–13.34)
Formal source^b^	Crude OR (95% CI)	1.87 (1.02–3.72)	3.69 (2.01–7.34)
	aOR (95% CI)	1.68 (0.83–3.71)	4.41 (2.16–9.89)
Informal source^b^	Crude OR (95% CI)	2.17 (1.02–5.35)	5.24 (2.49–12.85)
	aOR (95% CI)	2.35 (0.96–7.08)	6.34 (2.61–19.06)
In last 4 weeks			
Any source	Crude OR (95% CI)	2.69 (1.05–9.11)	4.84 (1.89–16.38)
	aOR (95% CI)	2.55 (0.84–11.13)	6.02 (1.97–26.43)
Formal source	Crude OR (95% CI)	2.45 (0.84–10.39)	4.08 (1.40–17.32)
	aOR (95% CI)	2.36 (0.62–15.62)	6.58 (1.71–44.07)
Informal source	Crude OR (95% CI)	1.93 (0.65–8.31)	3.63 (1.24–15.52)
	aOR (95% CI)	2.02 (0.53–13.33)	4.93 (1.29–32.64)
**Year 3**			
In the last 12 months			
Any source	Crude OR (95% CI)	1.58 (0.92–2.87)	2.70 (1.55–4.94)
	aOR (95% CI)	1.38 (0.76–2.59)	2.27 (1.24–4.32)
Formal source	Crude OR (95% CI)	1.40 (0.76–2.75)	2.75 (1.49–5.41)
	aOR (95% CI)	1.03 (0.53–2.13)	2.34 (1.20–4.84)
Informal source	Crude OR (95% CI)	1.87 (0.90–4.37)	2.96 (1.42–6.97)
	aOR (95% CI)	1.70 (0.78–4.16)	2.14 (0.96–5.32)
In the last 4 weeks			
Any source	Crude OR (95% CI)	1.00 (0.38–3.09)	1.90 (0.74–5.82)
	aOR (95% CI)	1.11 (0.50–2.77)	1.88 (0.83–4.72)
Formal source	Crude OR (95% CI)	1.82 (0.68–6.29)	3.31 (1.26–11.42)
	aOR (95% CI)	1.22 (0.43–4.45)	2.84 (0.99–10.38)
Informal source	Crude OR (95% CI)	1.09 (0.46–3.02)	1.90 (0.80–5.26)
	aOR (95% CI)	0.95 (0.37–2.77)	1.36 (0.52–4.00)

a For Year 1: formal source includes GP, hospital, therapist, NHS 111; informal source includes voluntary/charity and family/friends.

b For Years 2 and 3: formal source includes GP, nurse, mental health services and other health professional; informal source includes voluntary groups and family/friends.

c Adjusted odds ratios (aORs) adjusted for *a priori* confounders: age, sex, ethnicity, marital status, employment status, education level, previous illness, quintile of deprivation and local authority.

In the second year after flooding, when compared to unaffected individuals, flooded (aOR: 6.24, 95% CI: 3.18–13.34) and disrupted (aOR: 2.22, 95% CI: 1.14–4.68) individuals had greater odds of seeking help from any source in the previous 12 months. Flooded participants had the highest odds than unaffected participants of seeking help from both informal sources (aOR: 6.34, 95% CI: 2.61–19.06) and formal sources (aOR: 4.41, 95% CI: 2.16–9.89).

In the third year after flooding, flooded (aOR 2.27, 95% CI: 1.24–4.32) individuals continued to have greater odds of help-seeking behaviour than unaffected individuals, although to a lesser extent than the previous year. Disrupted individuals only showed slightly greater odds of help-seeking behaviour (aOR 1.38, 95% CI: 0.76–2.59) than unaffected individuals. Flooded individuals also had greater odds of receiving treatment in the second year after flooding compared to unaffected individuals, but this decreased in the third year after flooding (Supplementary table SA3).

## Discussion

### Summary

This study is the first to explore the impact of exposure to flooding on help-seeking behaviour in England over time. People whose homes were flooded or whose lives were disrupted by flooding had greater odds of seeking help from any source, and substantially increased odds of seeking help from informal sources, including the voluntary sector and family or friends, in the first year after flooding. This trend persisted in the second and third year after flooding, with flooded and disrupted participants having greater odds of seeking help from any source for mental health issues. Additionally, help-seeking behaviour from formal and informal sources was more prevalent in individuals with probable depression, anxiety or PTSD than those without a mental health outcome in the first, second and third year after flooding.

Importantly, this study has shown that the odds of seeking help from any source were significantly higher for both disrupted and flooded participants at 1 and 2 years after exposure to floods, and for flooded participants 3 years after exposure, indicating that demand for support can persist for up to 3 years after a flooding event. Additionally, in the second and third years after flooding, a greater proportion of flooded and disrupted individuals reported receiving treatment than the proportion of unaffected individuals. However, in the second year after flooding, a smaller proportion of the flooded and disrupted individuals who sought help received treatment than unaffected individuals who sought help, but this trend did not persist in the third year after flooding. Whilst not all individuals who seek help might require treatment, these results suggest that there could be a greater unmet need for help amongst those affected by flooding.

The possibility of an unmet need for help is supported by our finding that a notable proportion of individuals with any mental health outcome did not report seeking help from any source in the previous 12 months: it is troubling that around 15% of people with a probable mental health disorder had not sought help for any reason in the first year since flooding, while 33% and 40% had not specifically sought help for an emotional problem in the second and third years.

Previous studies have found conflicting evidence on the impact of flooding on longer-term primary and secondary healthcare use. There is limited research describing help-seeking after flooding from non-healthcare or informal resources, such as the voluntary sector or family and friends. This study identifies informal help as a widely used source of support.

Research has explored help-seeking behaviours following other types of natural event. One study described that 66% of individuals who had experienced weather-related disasters in the Gulf Coast reported a high frequency of depression and/or anxiety symptoms, but only 39% saw a medical professional, which suggests a possible unmet need for mental health services similar to that seen in our study.^33^ Another study explored help-seeking in men affected by a major earthquake in Chile and found underuse of psychosocial services, for reasons such as lack of time or association of help-seeking with physical health problems only.^32^ More generally, help-seeking behaviour in the form of health service use has been found to be more prevalent in people with a common mental disorder, including depression or anxiety, than those without.^34^ However, a range of existing literature has identified a treatment gap between the proportion of individuals experiencing mental disorders and the proportion who receive treatment. One study estimated a treatment gap of 45.4% for depression and 62.3% for generalized anxiety disorder across Europe.^35^ Other work has estimated only a quarter of adults with high levels of distress, and a third of adults with a diagnosable mental disorder, seek professional help.^10^^,^^17^

Our analysis is consistent with the findings of prior research by identifying that probable psychological morbidity is the likely source of the increase in help-seeking associated with flooding, whilst suggesting that the need for psychological support after flooding may be greater than estimated from reported help-seeking behaviour.

### Strengths and limitations

Help-seeking behaviour in this study was self-reported and we were not able to validate the reports against health or social care records. It is possible that the results are subject to recall bias, as those affected by flooding may be more likely to remember seeking help in the aftermath of their home being flooded than those who were entirely unaffected by flooding. This could result in an overestimate of the odds of help-seeking for those affected by flooding.

This research was conducted on a population affected by flooding that was predominately White, middle-aged and affluent living in England. We cannot exclude limitations in the generalizability of the findings to other populations. Previous research has suggested that sex, age, ethnicity, income and employment status significantly affect an individual’s likelihood of contacting a primary care physician, their perceived need for mental health services and their attitude to help-seeking.^30^^,^^32^^,^^36–38^ As there is no register of people affected by flooding, it is not known how representative those who responded are from the total population affected. Additionally, it is possible that the provision and availability of community and voluntary services were greater following flooding.

In Years 2 and 3, the questions regarding receiving treatment (including medication, counselling or other treatment for a stress-related, mental or emotional problem) might not capture participants receiving informal help, which could underestimate the number of participants who received any kind of support. In Years 2 and 3, the questions regarding help-seeking behaviour only included help sought for stress-related, emotional or mental health problems, whereas in Year 1, the question referred to help-seeking generally. This likely led to help-seeking behaviour appearing much lower in Years 2 and 3 than Year 1. It is worth noting that our estimate that about half of participants with a probable mental health outcome sought help from formal sources is consistent with research suggesting that about half of people with a common mental disorder discussed their mental health with a GP in the past year.^34^ Additionally, the more specific reasons for help-seeking were not sought in the questionnaire. Therefore, it is not possible to attribute the increased odds of help-seeking to issues directly related to participants’ exposure to flooding, rather than to significant life events other than flooding. Our aim was to describe the help-seeking behaviour of individuals affected by flooding up to 3 years after exposure, which could lead to an additional demand for health and care services when compared to people unaffected by flooding. Previous works have demonstrated that prevalence of psychological morbidity is higher in people affected by flooding and that this can be detected 3 years after the flooding event.^3–5^ It is therefore not surprising that this increase in prevalence would be associated with a burden on health and voluntary services.

### Implications for policy

The National Study of Flooding and Health has identified an increased prevalence of psychological morbidity 3 years after flooding.^3–5^ This analysis identified an increased demand on primary care, counselling and voluntary services or family and friends for several years following flooding. We also identified that there is likely to be an unmet need and demand for mental health support in people affected by flooding. Therefore, it is important that GPs and commissioners of services in areas at risk of flooding are aware that there may be a continuing need for support for at least 3 years after flooding has occurred, and that a more proactive approach might be required to provide assistance to those in need.

It is likely that the mental health needs of those affected by flooding are major determinants of help-seeking behaviour, given the increased prevalence of psychological morbidity in those flooded or disrupted by flooding, and the increased help-seeking behaviour in those with psychological morbidity. Therefore, interventions addressing psychological morbidity should be paramount in any strategy to support those affected by flooding. Further work should be undertaken to validate these findings.

We demonstrate a sizeable burden on health services following flooding. Research suggests that the risk of flooding is growing across Europe to 2050 but there are effective ways to reduce this risk.^18^ Action taken to reduce flood risk therefore reduces the health risk and associated burden on services demonstrated in this study. This aligns with the need for action to reduce flood risk laid out in the UK Climate Change Risk Assessment 2022, which should be tackled in the UK National Adaptation Programme 2023.^39^^,^^40^

## Conclusion

Flooding is associated with increased help-seeking behaviour from a range of healthcare and voluntary services for at least 3 years. The increased need and demand for help following flooding is an important issue for local health and social care systems to consider in the development of climate change adaptation plans, flood response plans and the commissioning of services to support those affected by flooding, specifically, focusing on health and community services that can help prevent psychological morbidity and mitigate the long-term health impact on communities following flooding through early detection and intervention.

## Supplementary Material

ckad082_Supplementary_Data

## Data Availability

The data underlying this article may be requested from the corresponding author and applications will be reviewed in line with UKHSA policies and procedures.
