# Supplementary material for: Help-seeking following a flooding event: a cross-sectional analysis of adults affected by flooding in England in winter 2013/14
Source: Eur J Public Health. 2023 Jun 16;33(5):834–40. doi: 10.1093/eurpub/ckad082 (PMC10567239; doi:10.1093/eurpub/ckad082)
Supplement: ckad082_Supplementary_Data [file ckad082_supplementary_data.docx]

Supplementary Material

**Figure A1: Help seeking question from Year 1 questionnaire**


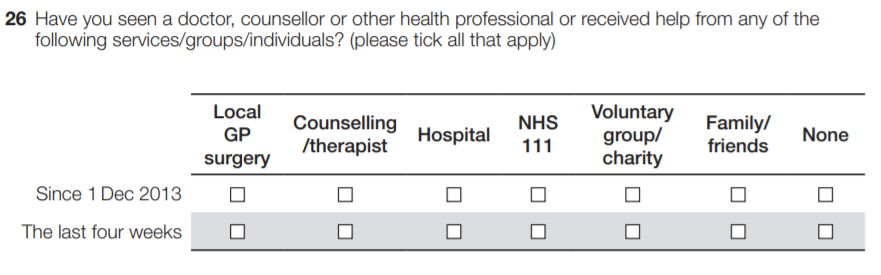


**Figure A2: Help seeking questions from Year 2 questionnaire**


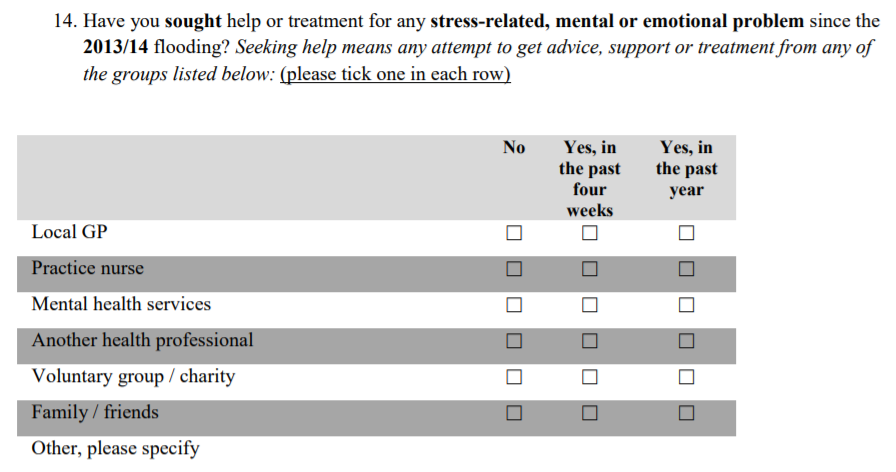


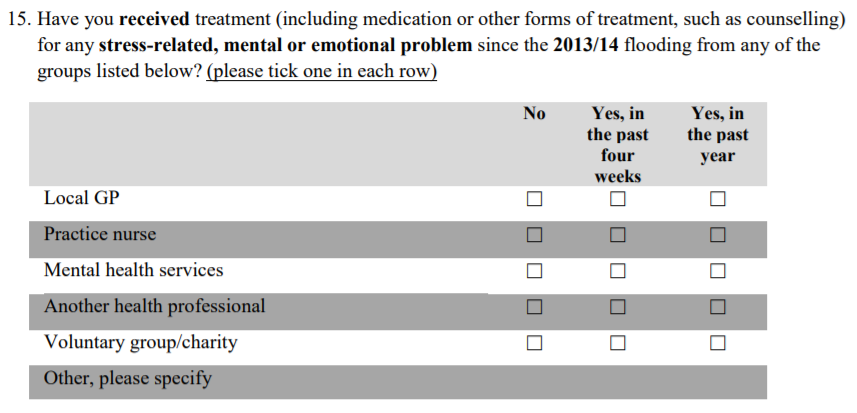


**Figure A3: Help seeking questions from Year 3 questionnaire**


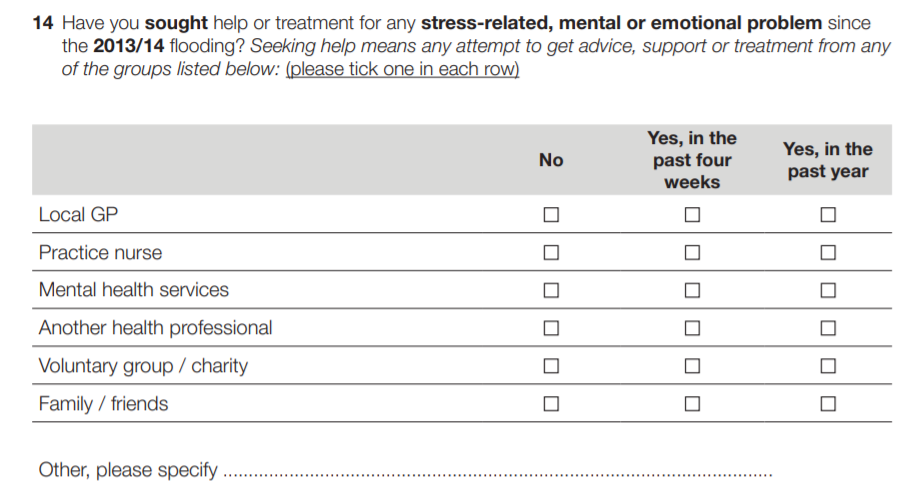


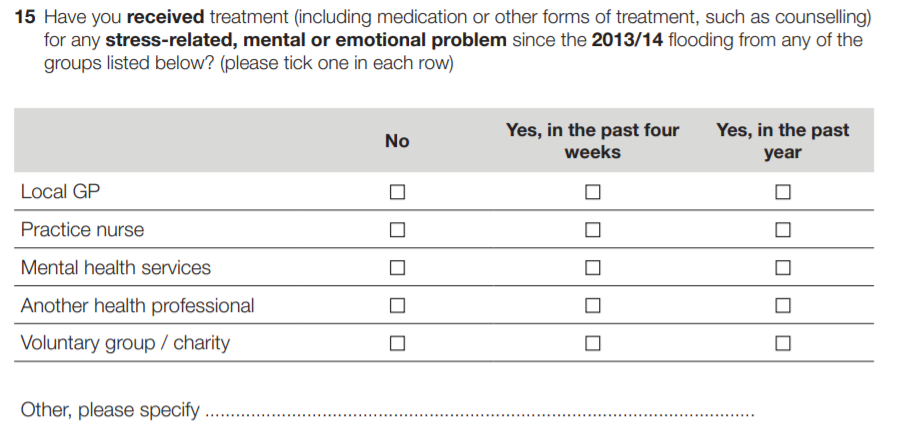


| **Table A1: Number of individuals who sought help by exposure status, with detailed breakdown of the type of help sought, Year 1, 2, and 3 (n (%))** | | | | |
| --- | --- | --- | --- | --- |
| **Outcome: Sought help** |  |  |  |  |
| **Year 1** | **Unaffected (n=285)** | **Disrupted (n=1099)** | **Flooded (n=622)** | **Overall (n=2006)** |
| **Since 1 Dec 2013** |  |  |  |  |
| GP | 153/280 (54.6) | 695/1060 (65.6) | 388/601 (64.6) | 1236/1941 (63.7) |
| Hospital | 66/280 (23.6) | 308/1060 (29.1) | 146/601 (24.3) | 520/1941 (26.8) |
| NHS 111 | 10/280 (3.6) | 45/1060 (4.2) | 25/601 (4.2) | 80/1941 (4.1) |
| Therapist | 8/280 (2.9) | 60/1060 (5.7) | 38/601 (6.3) | 106/1941 (5.5) |
| Voluntary/charity | 1/280 (0.4) | 24/1060 (2.3) | 38/601 (6.3) | 63/1941 (3.2) |
| Friends/family | 28/280 (10.0) | 225/1060 (21.2) | 189/601 (31.4) | 442/1941 (22.8) |
| None | 95/280 (33.9) | 236/1062 (22.3) | 144/604 (23.8) | 475/1946 (24.4) |
| **In the last 4 weeks** |  |  |  |  |
| GP | 77/280 (27.5) | 338/1060 (31.9) | 200/601 (33.3) | 615/1941 (31.7) |
| Hospital | 30/280 (10.7) | 155/1060 (14.6) | 75/601 (12.5) | 260/1941 (13.4) |
| NHS 111 | 3/280 (1.1) | 13/1060 (1.2) | 11/601 (1.8) | 27/1941 (1.4) |
| Therapist | 4/280 (1.4) | 35/1060 (3.3) | 24/601 (4.0) | 63/1941 (3.2) |
| Voluntary/charity | 1/280 (0.4) | 12/1060 (1.1) | 14/601 (2.3) | 27/1941 (1.4) |
| Friends/family | 25/280 (8.9) | 150/1060 (14.2) | 115/601 (19.1) | 290/1941 (14.9) |
| None | 133/280 (47.5) | 447/1062 (42.2) | 239/604 (39.8) | 819/1946 (42.1) |
| **Year 2** | **Unaffected (n=137)** | **Disrupted (n=512)** | **Flooded (n=339)** | **Overall (n=988)** |
| **In the last year** |  |  |  |  |
| GP | 8/134 (6.0) | 49/485 (10.1) | 59/326 (18.1) | 151/945 (16.0) |
| Nurse | 1/131 (0.8) | 6/460 (1.3) | 10/298 (3.4) | 33/889 (3.7) |
| Mental health services | 1/132 (0.8) | 9/460 (2.0) | 11/294 (3.7) | 30/886 (3.4) |
| Other health professional | 3/133 (2.3) | 14/461 (3.0) | 11/295 (3.7) | 48/889 (5.4) |
| Voluntary group | 2/132 (1.5) | 11/461 (2.4) | 14/294 (4.8) | 38/887 (4.3) |
| Family/friends | 3/130 (1.0) | 28/467 (6.0) | 43/296 (14.5) | 113/893 (12.7) |
| None | 121/134 (90.3) | 383/479 (80.0) | 205/323 (63.5) | 709/936 (75.4) |
| **In the last 4 weeks** |  |  |  |  |
| GP | 2/134 (1.5) | 17/485 (3.5) | 16/326 (4.9) | 35/945 (3.7) |
| Nurse | 1/131 (0.8) | 9/460 (2.0) | 6/298 (2.0) | 16/889 (1.8) |
| Mental health services | 2/132 (1.5) | 3/460 (0.7) | 4/294 (1.4) | 9/886 (1.0) |
| Other health professional | 1/133 (0.8) | 10/461 (2.2) | 9/295 (3.1) | 20/889 (2.2) |
| Voluntary group | 2/132 (1.5) | 4/461 (0.9) | 5/294 (1.7) | 11/887 (1.2) |
| Family/friends | 1/130 (0.8) | 18/467 (3.9) | 20/296 (6.8) | 39/893 (4.4) |
| None | 127/131 (96.9) | 425/461 (92.2) | 256/295 (86.8) | 808/887 (91.1) |
| **Year 3** | **Unaffected (n=119)** | **Disrupted (n=421)** | **Flooded (n=279)** | **Overall (n=819)** |
| **In the last year** |  |  |  |  |
| GP | 8/116 (6.9) | 52/405 (12.8) | 54/267 (20.2) | 114/788 (14.5) |
| Nurse | 3/114 (2.6) | 16/384 (4.2) | 17/249 (6.8) | 36/747 (4.8) |
| Mental health services | 4/114 (3.5) | 5/382 (1.3) | 20/252 (7.9) | 29/748 (3.9) |
| Other health professional | 4/114 (3.5) | 19/386 (4.9) | 20/251 (8.0) | 43/751 (5.7) |
| Voluntary group | 2/114 (1.8) | 7/384 (1.8) | 10/251 (4.0) | 19/749 (2.5) |
| Family/friends | 8/114 (7.0) | 46/389 (11.8) | 43/253 (17.0) | 97/756 (12.8) |
| None | 99/116 (85.3) | 316/402 (78.6) | 183/268 (68.3) | 598/786 (76.1) |
| **In the last 4 weeks** |  |  |  |  |
| GP | 0/116 (0.0) | 16/405 (4.0) | 17/267 (6.4) | 33/788 (4.2) |
| Nurse | 2/114 (1.8) | 7/384 (1.8) | 8/249 (3.2) | 17/747 (2.3) |
| Mental health services | 2/114 (1.8) | 0/382 (0.0) | 7/252 (2.8) | 9/748 (1.2) |
| Other health professional | 1/114 (0.9) | 9/386 (2.3) | 9/251 (3.6) | 19/751 (2.5) |
| Voluntary group | 2/114 (1.8) | 1/384 (0.3) | 6/249 (2.4) | 9/749 (1.2) |
| Family/friends | 5/114 (4.4) | 22/389 (5.7) | 22/253 (8.7) | 49/756 (6.5) |
| None | 106/114 (93.0) | 351/387 (90.7) | 214/252 (84.9) | 671/711 (94.4) |

| **Table A2: Number of individuals who received help by exposure status, with detailed breakdown of the type of help sought, Year 2 and 3 (n (%))** | | | | |
| --- | --- | --- | --- | --- |
| **Outcome: Received help** |  |  |  |  |
| **Year 2** | Unaffected (n=137) | Disrupted (n=512) | Flooded (n=339) | Overall (n=988) |
| **In the last year** |  |  |  |  |
| GP | 6/131 (4.6) | 48/485 (9.9) | 50/321 (15.6) | 104/937 (11.1) |
| Nurse | 3/129 (2.3) | 7/468 (1.5) | 5/294 (1.7) | 15/891 (1.7) |
| Mental health services | 3/129 (2.3) | 13/471 (2.8) | 16/298 (5.4) | 32/898 (3.6) |
| Other health professional | 4 /130 (3.1) | 15/472 (3.2) | 9/298 (3.0) | 28/900 (3.1) |
| Voluntary group | 2/130 (1.5) | 9/468 (1.9) | 8/295 (2.7) | 19/893 (2.1) |
| None | 123/132 (93.2) | 419/482 (86.9) | 253/314 (80.6) | 795/928 (85.7) |
| **In the last 4 weeks** |  |  |  |  |
| GP | 1/131 (0.8) | 12/485 (2.5) | 13/321 (4.0) | 26/937 (2.8) |
| Nurse | 1/129 (0.80) | 2/468 (0.4) | 1/294 (0.3) | 4/891 (0.4) |
| Mental health services | 2/129 (1.6) | 3/471 (0.6) | 6/298 (2.0) | 11/898 (1.2) |
| Other health professional | 2/130 (1.5) | 7/472 (1.5) | 3/298 (1.0) | 12/900 (1.3) |
| Voluntary group | 1/130 (0.8) | 4/468 (0.9) | 2/295 (0.7) | 7/893 (0.8) |
| None | 127/130 (97.7) | 447/470 (95.1) | 280/299 (93.6) | 854/899 (95.0) |
| **Year 3** | Unaffected (n=119) | Disrupted (n=421) | Flooded (n=279) | Overall (n=819) |
| **In the last year** |  |  |  |  |
| GP | 7/115 (6.1) | 37/403 (9.2) | 34/260 (13.1) | 78/778 (10.0) |
| Nurse | 2/114 (1.8) | 4/390 (1.0) | 10/250 (4.0) | 16/754 (2.1) |
| Mental health services | 2/114 (1.8) | 6/390 (1.5) | 18/255 (7.1) | 26/759 (3.4) |
| Other health professional | 3/114 (2.6) | 13/393 (3.3) | 15/252 (6.0) | 31/759 (4.1) |
| Voluntary group | 1/114 (0.9) | 6/390 (1.5) | 5/250 (2.0) | 12/754 (1.6) |
| None | 105/115 (91.3) | 351/398 (88.2) | 210/262 (80.2) | 666/775 (85.9) |
| **In the last 4 weeks** |  |  |  |  |
| GP | 2/115 (1.7) | 13/403 (3.2) | 8/260 (3.1) | 23/778 (3.0) |
| Nurse | 2/114 (1.8) | 3/390 (0.8) | 5/250 (2.0) | 10/754 (1.3) |
| Mental health services | 2/114 (1.8) | 1/390 (0.3) | 7/255 (2.7) | 10/759 (1.3) |
| Other health professional | 2/114 (1.8) | 5/393 (1.3) | 6/252 (2.4) | 13/759 (1.7) |
| Voluntary group | 1/114 (0.9) | 1/390 (0.3) | 3/250 (1.2) | 5/754 (0.7) |
| None | 109/114 (95.6) | 372/389 (95.6) | 230/250 (92.0) | 711/753 (94.4) |

| **Table A3: Crude and adjusted odds ratios (OR) of receiving treatment for disrupted or flooded, compared with unaffected respondents, Year 2 and 3.** | | | |
| --- | --- | --- | --- |
|  |  | Exposure group | |
| Outcome (received treatment) |  | Disrupted | Flooded |
| **Year 2** | Odds ratio (OR)** |  |  |
| **In the last 12 months** |  |  |  |
| Any source | Crude OR (95% CI) | 2.05 (1.04-4.54) | 3.30 (1.66-7.31) |
|  | aOR (95% CI) | 1.73 (0.81-4.18) | 3.43 (1.57-8.41) |
| Formal source* | Crude OR (95% CI) | 1.91 (0.96-4.22) | 3.08 (1.55-6.52) |
|  | aOR (95% CI) | 1.66 (0.78-4.02) | 3.14 (1.43-7.71) |
| Informal source* | Crude OR (95% CI) | 1.25 (0.32-8.30) | 1.78 (0.44-11.93) |
|  | aOR (95% CI) | 1.16 (1.72-2.33) | 3.19 (4.66-6.56) |
| **In the last 4 weeks** |  |  |  |
| Any source | Crude OR (95% CI) | 2.18 (0.74-9.29) | 2.87 (0.96-12.38) |
|  | aOR (95% CI) | 2.12 (0.57-13.81) | 3.15 (0.81-21.01) |
| Formal source | Crude OR (95% CI) | 1.78 (0.59-7.66) | 2.70 (0.89-11.68) |
|  | aOR (95% CI) | 1.85 (0.49-12.16) | 3.02 (0.76-20.30) |
| Informal source | Crude OR (95% CI) | 1.11 (0.16-21.86) | 0.88 (0.08-19.05) |
|  | aOR (95% CI) | NA | NA |
| **Year 3** |  |  |  |
| **In the last 12 months** |  |  |  |
| Any source | Crude OR (95% CI) | 1.41 (0.71-3.04) | 2.60 (1.32-5.61) |
|  | aOR (95% CI) | 0.99 (0.48-2.24) | 2.10 (1.00-4.76) |
| Formal source | Crude OR (95% CI) | 1.33 (0.68-2.89) | 2.60 (1.32-5.61) |
|  | aOR (95% CI) | 0.93 (0.44-2.09) | 2.07 (0.99-4.69) |
| Informal source | Crude OR (95% CI) | 1.77 (0.30-33.53) | 2.31 (0.37-44.46) |
|  | aOR (95% CI) | 0.82 (0.11-17.02) | 1.03 (0.12-22.07) |
| **In the last 4 weeks** |  |  |  |
| Any source | Crude OR (95% CI) | 1.00 (0.38-3.09) | 1.90 (0.74-5.81) |
|  | aOR (95% CI) | 0.56 (0.20-1.87) | 1.21 (0.42-4.07) |
| Formal source | Crude OR (95% CI) | 0.99 (0.38-3.08) | 1.79 (0.70-5.52) |
|  | aOR (95% CI) | 0.59 (0.20-1.95) | 1.19 (0.41-4.00) |
| Informal source | Crude OR (95% CI) | 0.29 (0.01-7.38) | 1.37 (0.17-27.91) |
|  | aOR (95% CI) | 0.16 (0.00-5.55) | 0.32 (0.02-9.28) |
| * For Year 2 and 3: formal source includes GP, nurse, mental health services and other health professional; informal source includes voluntary groups and family/friends  ** Adjusted odds ratios (aORs) adjusted for a priori confounders: age, sex, ethnicity, marital status, employment status, education level, previous illness, quintile of deprivation and local authority | | | |
